# *QuickStats:* Percentage* of Emergency Department Visits for Acute Viral Upper Respiratory Tract Infection^†^ at Which an Antimicrobial Was Given or Prescribed,^§^ by Age — United States, 2010–2017^¶^

**DOI:** 10.15585/mmwr.mm6906a6

**Published:** 2020-02-14

**Figure:**
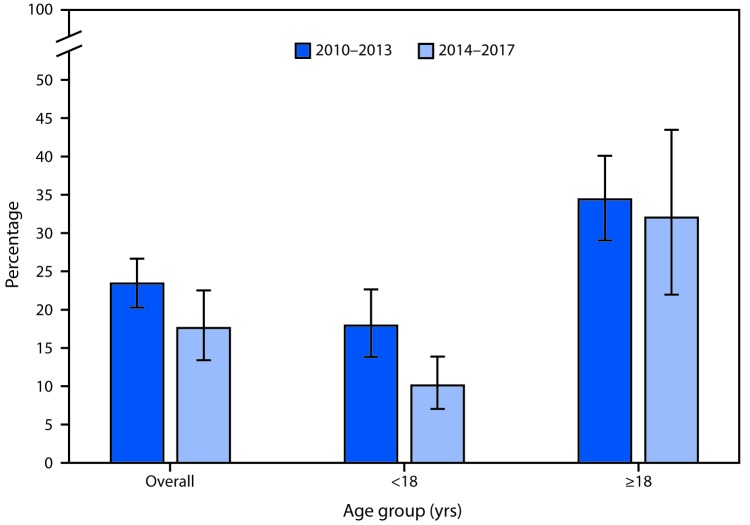
From 2010–2013 to 2014–2017, the percentage of emergency department (ED) visits for acute viral upper respiratory tract infection that had an antimicrobial given or prescribed, hereafter referred to as ED visits, decreased from 23.4% to 17.6%. A decline was also seen for ED visits by children, decreasing from 17.9% to 10.1%, but a decline was not seen for ED visits by adults. In both periods, the percentage of ED visits by adults was higher than the percentage of ED visits by children.

**Source:** National Center for Health Statistics. National Hospital Ambulatory Medical Care Survey, 2010–2017. ftp://ftp.cdc.gov/pub/Health_Statistics/NCHS/Datasets/NHAMCS.

